# Intravitreal anti-vascular endothelial growth factor and combined photodynamic therapy for pachychoroid neovasculopathy: long-term treatment outcomes

**DOI:** 10.1007/s00417-024-06387-z

**Published:** 2024-01-31

**Authors:** Nobuya Tanaka, Keiko Azuma, Shuichiro Aoki, Kohdai Kitamoto, Kohei Ueda, Ryosuke Fujino, Tatsuya Inoue, Ryo Obata

**Affiliations:** 1 Department of Ophthalmology, Graduate School of Medicine and Faculty of Medicine, The University of Tokyo Hospital, Tokyo, 113-8655 Japan; 2 Department of Ophthalmology, Shinseikai Toyama Hospital, Toyama, Japan; 3 Department of Ophthalmology, Teishin Hospital, Tokyo, Japan; 4 Department of Ophthalmology, Yokohama City University Medical Center, Yokohama, Japan

**Keywords:** Pachychoroid neovasculopathy, Photodynamic therapy, Intravitreal aflibercept, Treat and extend

## Abstract

**Purpose:**

To examine the long-term visual outcomes after initial treatment with combined photodynamic therapy (PDT) or aflibercept treat-and-extend (TAE) monotherapy in patients with pachychoroid neovasculopathy (PNV).

**Methods:**

Patients diagnosed with PNV, initially treated with PDT combined with anti-vascular endothelial growth factor (VEGF) or intravitreal aflibercept (IVA) monotherapy in the TAE protocol and followed up for at least 6 months, were included in the study. Medical records were retrospectively reviewed. Survival analysis was performed, in which deterioration in logMAR visual acuity by 0.1 or 0.3 is defined as “death.” The annual number of treatments was also analyzed. Sub-analysis was performed on 33 patients diagnosed with PNV without polypoidal lesions.

**Results:**

This study included 46 patients (23 in the initial combined PDT group and 23 in the IVA TAE group). Mean age, sex, mean baseline logMAR visual acuity, or duration of observation (3.6 ± 3.2 years vs. 3.1 ± 1.9 years) in both groups were comparable. As for visual outcome, no significant differences were found in survival analysis based on worsening of 0.1 or 0.3 logMAR (3-year survival; 26% vs. 26%, 91% vs. 90%, respectively). Meanwhile, the additional number of anti-VEGF injections per year was significantly lower in the initial combined PDT group than in the IVA TAE group (1.0 ± 1.3 vs. 4.1 ± 1.5, *p* < 0.0001). No significant differences were found in the number of additional PDTs per year (0.07 ± 0.20 vs. 0.02 ± 0.09, p = 0.27). Similar results were found in a sub-analysis of 33 patients without polyps.

**Conclusion:**

In the treatment of PNV, regardless of the presence of polyps, the long-term visual outcomes were similar between the initial combined PDT and IVA TAE monotherapy. However, the annual number of anti-VEGF injections was lower in the initial combined PDT group than in the aflibercept TAE group, whereas that of PDT was comparable.

**Supplementary Information:**

The online version contains supplementary material available at 10.1007/s00417-024-06387-z.


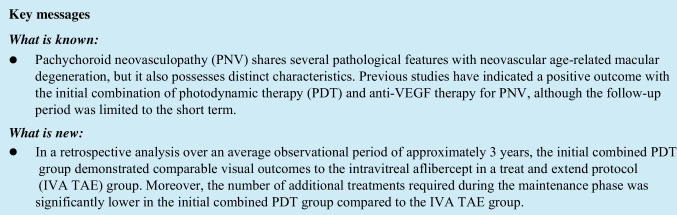


## Introduction

Recently, a condition known as pachychoroid neovasculopathy (PNV) has been reported, characterized by maculopathy with macular neovascularization (MNV) and exudative changes [1, 2]. PNV shares anatomic features with other pachychoroid diseases, such as choroidal thickening, increased choroidal permeability, MNV-independent pigment epithelial abnormalities, choroidal mid-major vessel dilation, and abnormal choroidal circulation, which may play an important role in the pathogenesis of PNV. PNV shares some findings with conventional age-related macular degeneration (AMD), such as drusen deposition, pigmentary abnormalities, and type 1 MNV. Exudative AMD with macular neovascularization (MNV) causes severe visual impairment and intravitreal injection of anti-vascular endothelial growth factor (VEGF) inhibitors or photodynamic therapy (PDT) are approved treatments for exudative AMD [3–8]. Moreover, anti-VEGF therapy combined with PDT has been proven to be effective for MNV with polypoidal lesions [9]. As for PNV, similar treatments such as anti-VEGF therapy and PDT have been used; however, the response to treatment differs from that of conventional AMD; that is, exudates are more likely to persist or recur in response to anti-VEGF treatment than non-PNV exudates [10, 11]. Regarding visual prognosis, improvement in visual acuity after anti-VEGF treatment is worse in patients with PNV [12, 13]. In contrast, anti-VEGF therapy combined with PDT is effective for PNV, with and without polyps [14–18]. However, these are 1-year follow-up reports for a single treatment group, and none has compared multiple treatments or examined long-term outcomes.

In this study, we conducted a retrospective analysis of various background factors, visual outcomes, and treatment frequency in patients who received a combination of anti-VEGF therapy with PDT or aflibercept TAE therapy for PNV for more than 3 years on average.

## Methods

### Study design

This retrospective study was performed in accordance with the tenets of the Declaration of Helsinki and was approved by the ethics committee of the University of Tokyo Faculty of Medicine. All data were fully authorized before access. Written informed consent was not required; nonetheless, participants who did not provide authorization to use their medical records for research were excluded from the study.

### Participants

This study included patients who were treated at the Department of Ophthalmology at the University of Tokyo Hospital between January 2011 and September 2022. These patients were diagnosed with PNV and received initial therapy consisting of a combination of anti-VEGF treatment with PDT or aflibercept TAE. All patients underwent a standard examination that included the measurement of best-corrected visual acuity (BCVA), slit-lamp biomicroscopy, fundoscopy, and spectral-domain optical coherence tomography (Spectralis; Heidelberg Engineering, Heidelberg, Germany) at each visit. BCVA was measured using the Landolt C chart, and the values were converted into the logarithm of the minimal angle of resolution (logMAR). All patients underwent fluorescein angiography and indocyanine green angiography (ICGA) at the time of initial treatment unless contraindicated (Heidelberg Retina Angiograph 2; Heidelberg Engineering, Heidelberg, Germany). The diagnostic criteria for PNV were as follows: (1) presence of choroidal vascular hyperpermeability (CVH). CVH is defined on ICGA as irregular areas of increasing fluorescence during the mid- and late-phases, often surrounding the dilated pachyvessels [19]; (2) detection of MNV using OCTA or fluorescence fundus angiography. Diagnoses were made by two ophthalmologists specializing in the macular region (K.A. and R.O.). The exclusion criteria were as follows: (1) less than 6 months of observation from the initiation of treatment and (2) accompanying diabetic retinopathy and retinal vein occlusion, including macular or other retinal diseases.

### Treatments

Because the patients were treated in the setting of real-world practice, the treatment for each patient was selected through the following process: for patients showing signs of pachychoroid, PDT in combination with anti-VEGF was presented as an alternative option distinct from anti-VEGF monotherapy. After explaining the details of each treatment, particularly focusing on the risk of photosensitivity in PDT and the necessary precautions, the physician finally determined the treatment considering the patient’s preferences.

Combination therapy consisted of a single injection of 0.5 mg ranibizumab or 2 mg aflibercept and standard-fluence PDT (administered using a 689-nm diode laser unit) (Visulas PDT system 690 S; Carl Zeiss AG, Oberkochen, Germany) following intravenous verteporfin (Visudyne; Novartis AG, Basel, Switzerland) in accordance with the guidelines for applying PDT in polypoidal choroidal vasculopathy [14]. Additional injections of aflibercept or ranibizumab were administered during monthly follow-up visits when reactivation of the MNV was suggested. In the initial combined PDT group, as needed anti-VEGF treatments were given if exudative changes were recognized under monthly follow-up. In both treatment groups, PDT was administered unless the most recent PDT has been undergone within 3 months, in case exudative changes persisted or frequently recurred.

Aflibercept TAE treatment consisted of three monthly injections of 2 mg aflibercept, followed by a maintenance phase in a TAE regimen. The protocol was based on the multicenter collaborative study demonstrating the efficacy of aflibercept TAE therapy for neovascular AMD in Japanese patients [6]. Briefly, in the maintenance phase, the interval between injections was extended by 4 weeks if there were no exudative changes suggesting MNV activity, whereas it was shortened by 4 weeks if they were found during follow-up. After shortening the initial interval, the subsequent extension interval was set to 2 weeks. The maximum interval was set to 16 weeks.

### Evaluation of macular atrophy

Macular atrophy (MA) was evaluated based on the SD-OCT images and color fundus photographs at baseline, year 1, year 2, year 3, and year 5, as previously reported [20, 21]. Briefly, the criteria were thinning of the RPE band on OCT images, increased signal transmission in the choroid on OCT images, and no contradicting findings on color fundus photographs. Additionally, a hyper-reflective area on infrared fundus photography was served as a reference. Two retina specialists (NT, RO) independently made the diagnosis. In cases with a disagreement between the two graders, the results were discussed until a consensus was reached.

### Statistical analysis

We reviewed the medical charts of the included patients on January 2023 or later. In patients who continued to be followed up with the same treatment protocol until the time of the chart review, the observation period was calculated as the time from the initial treatment to the chart review. On the other hand, the patients who discontinued the visit our clinic or had changed treatments before the chart review were considered dropouts. In the dropouts, the observation period was calculated as the time from the initial treatment to the last visit before discontinuation of the visit or change in the treatment. Presence or absence of remarkable visual loss or any events having possibility of detrimental visual loss was also assessed. Age, sex, baseline logMAR visual acuity, observation period, and number of cases with polyp foci on ICGA were compared between the initial combined PDT and IVA TAE groups. The change in the logMAR VA from baseline was analyzed and compared between the two treatment groups. A *t*-test was performed for continuous variables and a chi-square test for categorical variables. In the Kaplan–Meier survival analysis, “death” was defined as a decrease in visual acuity to 0.3 logMAR or worse or a worsening of visual acuity by 0.3 logMAR or 0.1 logMAR or greater from baseline. Log-rank tests were performed to compare the groups. The total number of anti-VEGF or PDT treatments per year was calculated by dividing the total number of treatments with the observation period. Similarly, the annual number of maintenance treatments per year was calculated using the number of treatments after excluding one IVA and one PDT in the initial combined PDT group or three IVA in the IVA TAE group from the total number of treatments. They were then compared between treatments using the Mann–Whitney *U* test. Similar analyses were performed between subgroups in which no polyps were found. JMP software (version 16, SAS Institute) was used for the analysis and was considered statistically significant at *p* < 0.05. For multiple comparisons, the Bonferroni correction of *p*-values or Tukey’s HSD test was performed.

## Results

We included 46 eyes of 46 patients (23 in the initial combined PDT group and 23 in the IVA TAE group). There were no significant differences between the two groups in terms of mean age, sex, baseline logMAR visual acuity, or percentage of cases with polyps (Table 1).

**Table 1:** Background factors of the initial combined PDT group and IVA TAE group. *PDT*, photodynamic therapy; *IVA*, intravitreal aflibercept; *TAE*, treat and extend

	Initial combined PDT group	IVA TAE group	*p* value
Number	23	23	
Mean age	68.3 ± 9.53	67.7 ± 9.78	0.84
[Range]	[47, 85]	[51, 85]	
Women/men	5/18	6/17	0.73
Polyp ( −)/( +)	16/7	17/6	0.82
Initial logMAR visual acuity			
Mean ± SD	0.22 ± 0.23	0.19 ± 0.28	0.66
[Range]	[− 0.08, − 0.70]	[− 0.08, − 1.00]	
Follow-up period (months)			
Mean ± SD	43.4 ± 38.6	36.7 ± 22.6	0.48
[Range]	[7–142]	[6–82]	

Among 46 eyes included in the analysis, 42 (91%), 31 (67%), or 20 (44%) eyes had an observation period longer than 1, 2, or 3 years, respectively. Four out of 46 eyes were considered dropouts. Two of these 4 eyes were in the initially combined PDT group, and discontinued their visits at the fourth or the fifth year. The other 2 eyes were in the IVA TAE group and discontinued their visits at the second or the third year. All four eyes did not have remarkable visual loss or any events having possibility of detrimental visual loss until the last visit. The other 42 eyes continued their visits with the same treatment protocol until the time of the chart review. The observation time of these 42 eyes was calculated as the time from the initial treatment to the chart review, and 38 eyes, 28 eyes, and 18 eyes had the observation period longer than 1 year, 2 years, and 3 years, respectively. Combining these dropouts and non-dropouts, among totally 46 eyes at baseline, 42 (91%), 31 (67%), or 20 (44%) eyes had an observation period longer than 1, 2, or 3 years, respectively, and were used for the following analyses. The mean observation periods for the overall (46 eyes), initial combined PDT (23 eyes), and IVA TAE (23 eyes) group were 40.0, 43.4, and 36.7 months, respectively.

Overall, the mean logMAR VA at baseline and at 1, 2, and 3 years were 0.204 (46 eyes), 0.126 (42 eyes), 0.130 (31 eyes), and 0.144 (20 eyes), respectively. The mean logMAR VA at the final visit was 0.178 (46 eyes). The changes in the mean logMAR VA from baseline at 1, 2, and 3 years were − 0.080, − 0.071, and − 0.079, respectively. Visual acuity was comparable to baseline at 1, 2, and 3 years (*p* = 0.055, 0.132, and 0.117, respectively, with Tukey’s HSD test; Online Resource 1). Mean logMAR visual acuity was not significantly different between the two treatment groups at any time point (*p* = 0.660, 0.560, 0.530, and 0.948, Online Resource 2).

**Fig. 1:**
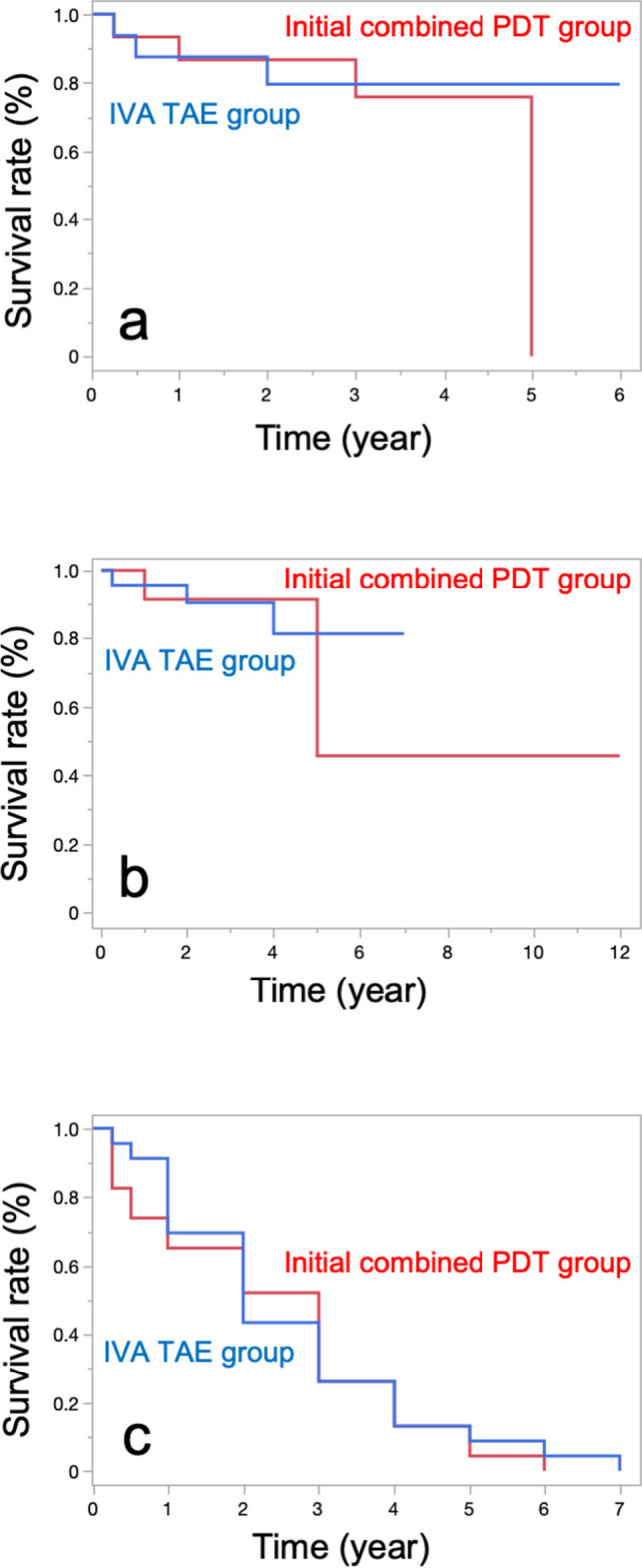
Survival analysis for the initial combined PDT and IVA TAE groups. In each graph, death was defined as **a** logMAR acuity > 0.3, **b** logMAR acuity change > 0.3, and **c** logMAR acuity change > 0.1, respectively. No significant differences were found between the two groups. LogMAR, logarithm of the minimal angle of resolution; PDT, photodynamic therapy; IVA, intravitreal aflibercept; TAE, treat and extend

In a survival analysis in which logMAR VA ≥ 0.3, logMAR VA change ≥ 0.3, or logMAR VA change ≥ 0.1 was defined as death, the 3-year survival rates in the initial combined PDT and IVA TAE groups were 75.8% and 79.6%, 91.3% and 90.3%, or 26.1% and 26.1%, respectively. There were no significant differences between the two groups in the log-rank test (Fig. 1 and Online Resource 3).

The mean number of additional anti-VEGF injections, excluding one PDT and one IVT in the initial combined PDT group and three IVAs in the IVA TAE group, was 1.00 per year in the initial combined PDT group and significantly fewer than 4.12 per year in the IVA TAE group (*p* < 0.0001, Table 2). The mean number of additional PDT sessions was 0.071 per year in the initial combined PDT group and 0.019 per year in the IVA TAE group, with no significant difference between the two groups (*p* = 0.271, Table 2).

**Table 2:** Annual average number of additional anti-VEGF injections and PDTs, excluding the first PDT/IVT in the initial combined PDT group and the three IVA induction cycles in the IVA TAE group

	Initial combined PDT group	IVA TAE group	*P* value
No. of cases	23	23	
Anti-VEGF injections (times/year)	1.0 ± 1.3	4.1 ± 1.5	*p* < 0.0001
PDTs (times/year)	0.07 ± 0.20	0.02 ± 0.09	*p* = 0.27

At baseline, one eye of each group had macular atrophy not involving the fovea. Then, 0/23 (0%), 2/22 (9%), 2/16 (13%), and 2/5 (40%) of the patients in the initial combined PDT group showed newly developed atrophy at year 1, year 2, year 3, and year 5. On the other hand, 3/23 (13%), 3/18 (17%), 1/14 (7%), and 1/7 (14%) of the patients in the IVA TAE group presented newly developed atrophy at year 1, year 2, year 3, and year 5. Among them, fovea-involving atrophy was developed only in the 2 eyes in the initial combined PDT group. There was no statistical difference in the incidence of atrophy between the two groups at each period.

The same analysis was performed for subgroups that consisted of only patients without polyps.

The background factors included the number of eyes, age, sex, logMAR VA at baseline, and duration of observation, with no significant differences (Online Resource 4).

Overall, the mean logMAR VA at baseline and at 1, 2, and 3 years were 0.201 (33 eyes), 0.130 (29 eyes), 0.147 (23 eyes), and 0.171 (13 eyes), respectively. The mean logMAR VA at the final visit was 0.179 (33 eyes). The mean logMAR VA changes from baseline at 1, 2, and 3 years were − 0.073, − 0.039, and 0.051, respectively. Visual acuity was comparable to baseline at 1, 2, and 3 years (*p* = 0.218, 0.432, and 0.394, respectively, with Tukey’s HSD test). The mean logMAR VA in each period was not significantly different between the two treatment groups (*p* = 0.936, 0.941, and 0.231, respectively, Online Resource 5).

In a survival analysis in which logMAR VA ≥ 0.3, logMAR VA change ≥ 0.3, or logMAR VA change ≥ 0.1 was defined as death, the 3-year survival rates in the initial combined PDT and IVA TAE groups were 71.4% and 68.2%, 87.5% and 86.3%, or 18.8% and 11.8%, respectively. There were no significant differences between the two groups in the log-rank test (Online Resource 6, 7, 8, and 9).

The mean number of additional anti-VEGF injections, excluding one PDT and one IVT in the initial combined PDT group and excluding three IVAs in the IVA TAE group, was 0.87 per year in the initial combined PDT group, which was significantly fewer than 4.37 per year in the IVA TAE group (*p* < 0.0001; Online Resource 10). The mean number of additional PDT sessions was 0.062 per year in the initial combined PDT group and 0.026 per year in the IVA TAE group, with no significant difference between the two groups (*p* = 0.550, Online Resource 10).

Also, we performed a sub-analysis to compare outcomes between the eyes with polyps and those without polyps in each treatment group. In both groups, there was no significant difference in the mean logMAR VA at year 1, year 2, and year 3, survival analysis with abovementioned three threshold, or treatment number of additional anti-VEGF or PDT between the eyes with polyps and those without polyps.

## Discussion

This retrospective study included 46 eyes that were diagnosed with PNV. Patients were divided into two groups: initial combined PDT and IVA TAE, with no significant differences in background factors for comparison of visual outcomes and treatment numbers. There was no statistical difference in the visual outcomes between the groups. However, the mean annual number of additional anti-VEGF injections during the course of the study was significantly lower in the initial combined PDT group than in the IVA TAE group, whereas the mean annual number of additional PDTs was comparable. These findings were similar when patients were restricted to those without polyps.

In the current study, there was no significant difference in the visual outcomes between the initial combined PDT and IVA TAE groups. The mean baseline VA for all PNV cases was 0.204, that was similar to the baseline VA of previous studies that investigated treatment-naïve patients with PNV [11, 17, 22]. The baseline VA was consistent with that in the current analysis. The results of the current analysis showed that both groups maintained comparable visual outcomes at 1–3 years. This suggests that IVT with initial combined PDT and IVA TAE for PNV could show comparable visual outcomes, which may not change over 3 years. However, some patients in the present study were lost to follow-up; thus, we added a survival analysis to compare the visual outcomes between the two treatments. The results also showed no significant difference between treatments when considering “death” as a decrease in VA to 0.3 logMAR or worse, a worsening of VA by 0.3 logMAR or greater, or a worsening of VA by 0.1 logMAR or greater, with mean observation periods exceeding 3 years. Although prospective studies with a larger number of patients are needed, the visual outcomes in approximately 3 years should be comparable between the initial combined PDT and IVA TAE.

However, there was a significant difference in the number of treatments between the two groups. In the initial combined PDT group, the mean annual number of additional IVT and PDT were 1.00 and 0.07, respectively. The results were comparable to those of a previously reported short-term study for initial combined PDT [17], where the mean number of additional IVT was 0.9 and that of additional PDT was 0.1 in a year. In the IVA TAE group of the current study, meanwhile, the mean annual number of additional IVT and PDT was 4.12 and 0.02, respectively. This number appears smaller than that in a previous study for IVA TAE, where the total number of additional treatments was 19.2 in 2 years [11], probably because in the current cohort, PDT was combined if the activity of the lesion persisted despite repeated IVA. Nevertheless, the number of IVT in the initial combined PDT group was significantly lower than that in the IVA TAE group in the present study. Although there should be some bias because of the retrospective nature of the present analysis, the results suggest that the initial combined PDT was associated with a decrease in the number of additional IVT at least within 3 years. Pachychoroid-related circulatory disturbances, such as choroidal congestion and increased capillary permeability, are thought to modulate the development and activity of neovascularization [1]. PDT produces temporary occlusion at the depth of the choroidal capillaries, resulting in a long-term decrease in choroidal thickness [23]. Such vascular remodeling due to PDT might, at least partly, contribute to the fact that combined PDT for PNV allows the control of PNV activity, leading to fewer anti-VEGF injections during the maintenance phase.

Furthermore, there was no significant difference in the number of additional PDT sessions between the initial combined PDT and IVA TAE groups. Considering that choroidal thickness reduction with PDT has been reported to be relatively long-lasting [23], the initial PDT may have played a role in the fact that the patients in this study did not require many additional PDT sessions within the observation period.

There was no difference in the visual outcomes between the two treatment groups, even when limited to patients without polypoidal lesions. This result is comparable to that of the 1-year outcome reported by Miki et al. [17]. Also, the visual outcomes and treatment number were similar between eyes with polyps and those without polyps in each treatment group. PDT has shown efficacy in terms of visual outcome and treatment number for PCV and AMD subtypes with polyps, as demonstrated in the EVEREST II randomized clinical trial [24]. However, recurrent exudation from MNV with or without polyps has been reported in the long term after PDT [25], suggesting that the long-term outcomes of PDT for PCV should not be determined by the presence/absence of polyp lesions. The results of the current study also showed that over a period of at least 3 years, there were no significant differences in visual outcomes or treatment requirements between patients with PNV with polyps and those without polyps. Although further studies with larger sample sizes are required, the effectiveness of combined PDT for PNV may be regulated by the pathology of the pachychoroid, rather than by the presence or absence of polyps.

In the present study, macular atrophy developed in some patients of each group with a comparable incidence. Yoon et al. reported that 3-year incidence of macular atrophy was 9.8%, that was smaller than typical AMD [26]. The result of the present study showed similar incidence (7%). Because thin choroid is a risk factor of development of atrophy [27], it may be rational to think that pachychoroid is protective against the formation of atrophy. Additionally, no statistical significance was found in the patients of the initial combined PDT group compared with the IVA TAE group through year 1 to year 5. There was little information on the incidence of atrophy after PDT for PNV. As for PCV, Miyakubo et al. mentioned that the frequencies of macular atrophy expansion in combination therapy during 2 years was 33% with no significant difference compared to anti-VEGF monotherapy (26%) [28]. However, Miyata et al. reported that 5-year incidence of macular atrophy was greater in initial combined PDT than in anti-VEGF monotherapy [21]. Considering the limited number of patients and retrospective nature of the current study, prospective study with larger samples should be necessary to elucidate whether or not the impact of these two treatments on the development of atrophy is distinct.

The current study had several limitations. First, this was a retrospective, single-center study. Second, the number of cases has decreased over time, and the number of cases that have been followed up for more than 3 years is also small. In addition, the official diagnostic criteria for PNV are unclear; some reports refer to PNV without distinguishing between cases with and without polyps, whereas others distinguish cases with polyps as PCV. In other respects, the type of IVT is not standardized in the initial combined PDT group; it includes a combination of IVA and IVR treatments. Furthermore, the indications for this study were limited to the Japanese population; therefore, further investigation is warranted to determine the applicability of the results to other ethnic groups.

In conclusion, based on the findings of this study, initial combined PDT with anti-VEGF therapy for PNV demonstrates comparable visual outcomes to IVA TAE therapy, particularly within a 3-year time frame. Furthermore, the combined therapy approach may require fewer treatment sessions. Similar conclusions can be drawn from the analysis of PNV cases with or without polyps. In terms of reducing the treatment and financial burden, this combination therapy may be one of the choices for treating eyes with PNV. However, further research is needed to strengthen the results of this study owing to the small number of cases and other biases.

### Supplementary Information

Below is the link to the electronic supplementary material.

Supplementary file1 (PDF 256 KB)
