# Supplementary material for: Intravitreal anti-vascular endothelial growth factor and combined photodynamic therapy for pachychoroid neovasculopathy: long-term treatment outcomes
Source: Graefes Arch Clin Exp Ophthalmol. 2024 Jan 31;262(6):1811–8. doi: 10.1007/s00417-024-06387-z (PMC11106190; doi:10.1007/s00417-024-06387-z)
Supplement: Supplementary file 1 — Supplementary file1 (PDF 256 KB) [file 417_2024_6387_MOESM1_ESM.pdf]

Intravitreal anti-vascular endothelial growth factor and combined photodynamic therapy for pachychoroid neovasculopathy; long-term treatment outcome.

For
Graefe's Archive for Clinical and Experimental Ophthalmology

Nobuya Tanaka[1,2], Keiko Azuma[1], Shuichiro Aoki[1], Kohdai Kitamoto[1], Kohei Ueda[1,3], Ryosuke Fujino[1], Tatsuya Inoue[4], Ryo Obata[1]

1) Department of Ophthalmology, The University of Tokyo Hospital, Tokyo, Japan
2) Department of Ophthalmology, Shinseikai Toyama Hospital, Toyama, Japan
3) Department of Ophthalmology, Teishin Hospital, Tokyo, Japan
4) Department of Ophthalmology, Yokohama City University Medical Center, Japan

Corresponding author:
Ryo Obata, MD, PhD
Department of Ophthalmology, Graduate School of Medicine and Faculty of Medicine, The University of Tokyo, Tokyo 113-8655, Japan
robata-tky@umin.ac.jp

ONLINE RESOURCES

Online Resources 1. Visual acuity progress in all cases. BL: baseline. *Compared with the logMAR VA at baseline. P-values were calculated using Tukye's HSD test.

	BL	1Y	2Y	3Y
Number	46	42	31	20
LogMAR VA	0.204	0.126	0.13	0.143
LogMAR VA change from BL		-0.08	-0.071	-0.079
P value*		0.055	0.132	0.117

Online Resources 2. Visual outcomes of the initial combined PDT and IVA TAE groups. PDT: photodynamic therapy. IVA: intravitreal aflibercept. TAE: treat and extend.

	BL	1Y	2Y	3Y
Initial combined PDT group				
LogMAR VA	0.22	0.146	0.159	0.14
No. of eyes	23	22	16	9
IVA TAE group				
LogMAR VA	0.188	0.104	0.098	0.147
No. of eyes	23	20	15	11
P value	0.66	0.56	0.53	0.95

Online Resources 3. Survival analysis with logMAR visual acuity >0.3, logMAR visual acuity change >0.3, and logMAR visual acuity change >0.1 as death for the initial combined PDT and IVA TAE groups. PDT: photodynamic therapy. IVA: intravitreal aflibercept. TAE: treat and extend.

	Initial combined PDT group		IVA TAE group	
	Survival rate(%)	No. at risk	Survival rate(%)	No. at risk
LogMAR>0.3				
1Y	87	14	88	14
2Y	87	12	80	11
3Y	76	8	80	8
4Y	76	4	80	6
P value			0.60	
LogMAR change >0.3				
1Y	91	23	96	22
2Y	91	20	90	18
3Y	91	14	90	13
4Y	91	8	81	10
P value			0.63	
LogMAR change >0.1				
1Y	65	17	74	21
2Y	52	15	43	16
3Y	26	12	26	10
4Y	13	6	13	6
P value			0.72	

Online Resources 4. Background Factors of Initial combined PDT group and IVA TAE group without polyps. No significant differences were found between the two groups for each item.

	Initial combined PDT group	IVATAE	P value
Number of cases	16	17	
Age			
Mean±SD	67.7±10.7	68.4±9.6	0.85
[Range]	[47-85]	[51-82]	
Gender			0.47
Women	3	5	
Men	13	12	
Initial logMAR Visual Acuity			
Mean±SD	0.18±0.206	0.221±0.293	0.64
[Range]	[-0.079 - 0.699]	[-0.079 - 1]	
Follow-up period (month)			
Mean±SD	37±28.1	37.8±25.3	0.93
[Range]	[7-111]	[6-82]	

Online Resources 5. Visual outcomes of the initial combined PDT and IVA TAE groups without polyps. No significant differences were found between the two groups.

	BL	1Y	2Y	3Y
Initial combined PDT group				
LogMAR VA	0.18	0.127	0.142	0.077
No. of eyes	16	15	12	6
IVA TAE group				
LogMAR VA	0.221	0.134	0.152	0.252
No. of eyes	17	14	11	7
P value	0.64	0.94	0.94	0.23

Online Resources 6: Survival analysis with logMAR visual acuity >0.3, logMAR visual acuity change >0.3, and logMAR visual acuity change >0.1 as death for the initial combined PDT and IVA TAE groups without polyps. PDT: photodynamic therapy. IVA: intravitreal aflibercept. TAE: treat and extend.

	Initial combined PDT group		IVA TAE group	
	Survival rate(%)	No. at risk	Survival rate(%)	No. at risk
LogMAR>0.3				
1Y	83	11	82	9
2Y	83	9	68	6
3Y	71	7	68	4
4Y	71	3	68	2
P value			0.77	
LogMAR change >0.3				
1Y	88	16	94	16
2Y	88	13	86	12
3Y	88	10	86	9
4Y	88	5	72	6
P value			0.77	
LogMAR change >0.1				
1Y	63	12	59	15
2Y	50	10	35	10
3Y	19	8	12	6
4Y	0	3	12	6
P value			0.96	

Online Resources 7. Survival analysis with logMAR acuity >0.3 as death for the initial combined PDT and IVA TAE groups without polyps. No significant differences were found between the two groups.

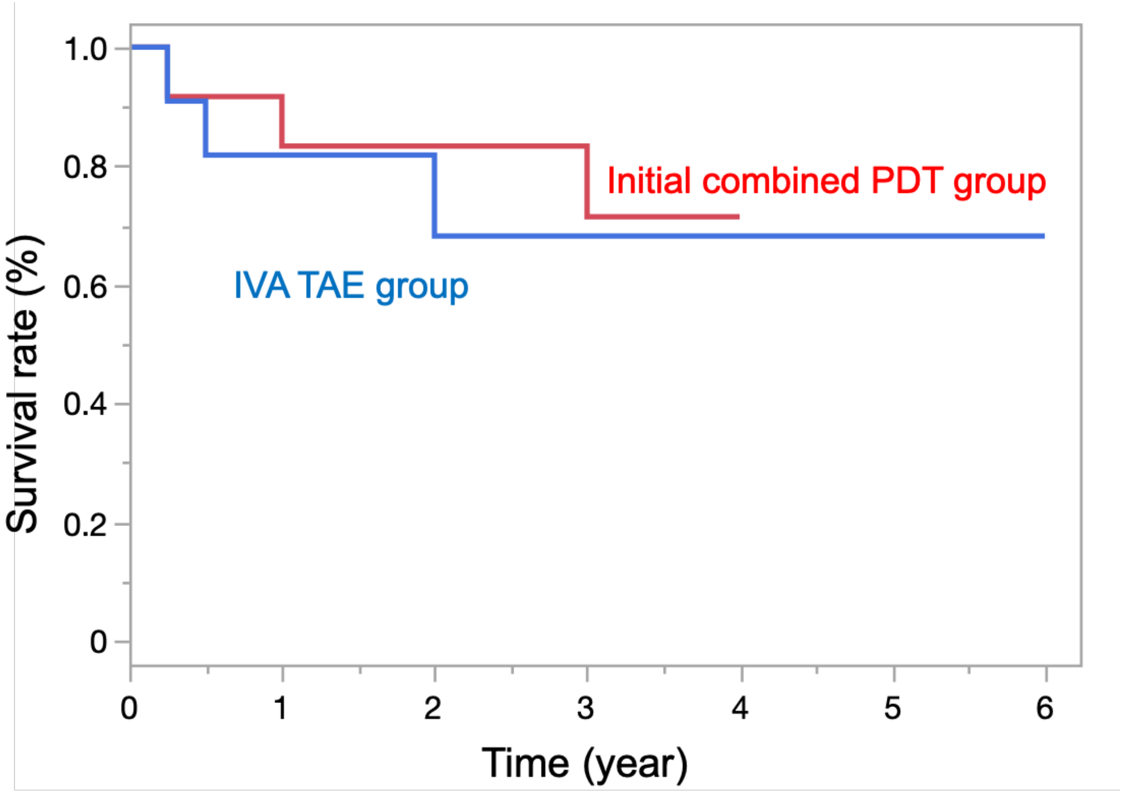

Online Resources 8. Survival analysis with logMAR acuity change >0.3 as death for the initial combined PDT and IVA TAE groups without polyps. No significant differences were found between the two groups.

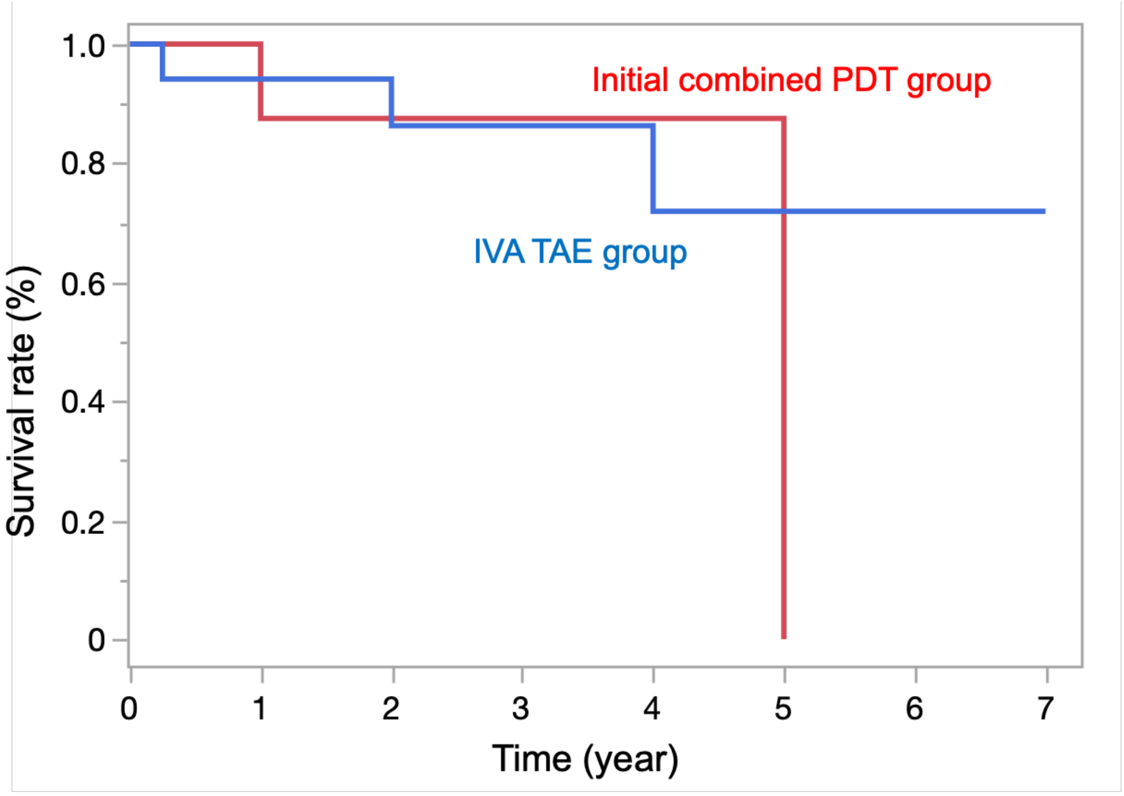

Online Resources 9. Survival analysis with logMAR acuity change >0.1 as death for the initial combined PDT and IVA TAE groups without polyps. No significant differences were found between the two groups.

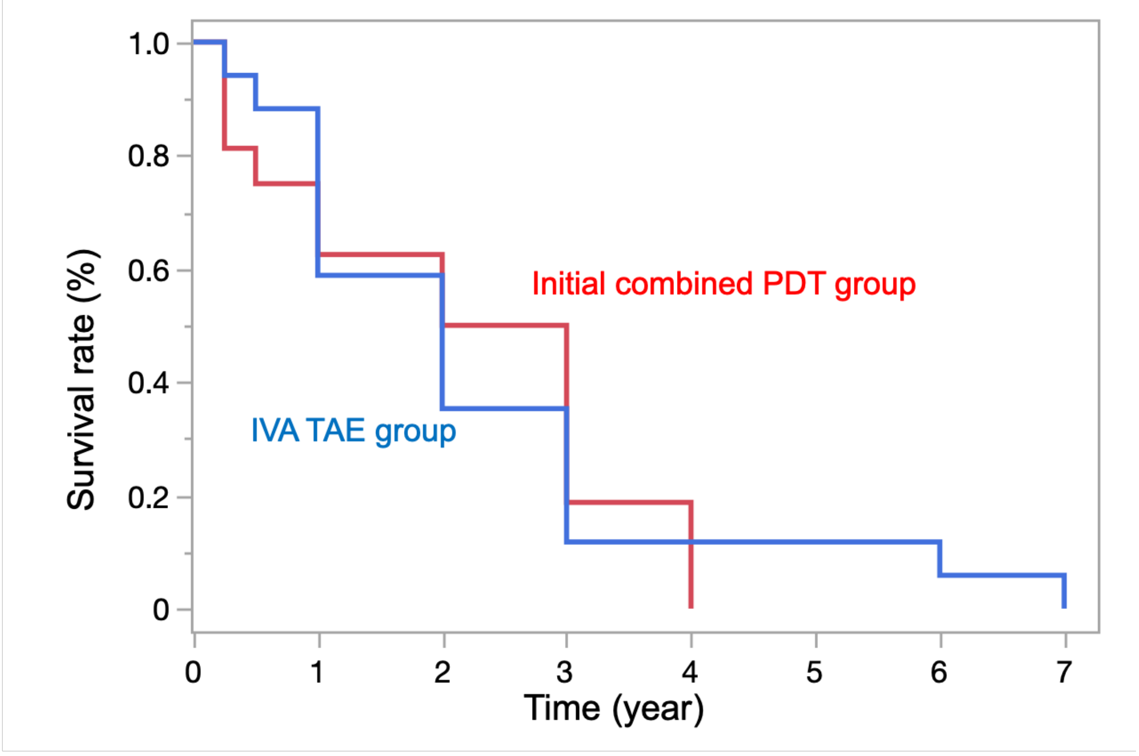

Online Resources 10. Annual average number of additional anti-VEGF injections and PDTs, excluding the first PDT/IVT in the initial combined PDT group and the three IVA induction cycles in the IVA TAE group.

	Initial combined PDT group	IVA TAE group	P value
No. of patients	16	17	
Anti-VEGF injection (times/year)	0.87±1.17	4.37±1.71	p<0.0001
PDT (times/year)	0.062±0.215	0.026±0.108	p=0.550
